# Modification of the synaptic cleft under excitatory conditions

**DOI:** 10.3389/fnsyn.2023.1239098

**Published:** 2023-09-28

**Authors:** Jung-Hwa Tao-Cheng, Sandra L. Moreira, Christine A. Winters, Thomas S. Reese, Ayse Dosemeci

**Affiliations:** ^1^NINDS Electron Microscopy Facility, National Institute of Neurological Diseases and Stroke, National Institutes of Health, Bethesda, MD, United States; ^2^Laboratory of Neurobiology, National Institute of Neurological Diseases and Stroke, National Institutes of Health, Bethesda, MD, United States

**Keywords:** synaptic cleft, electron microscopy, transsynaptic bridge, synaptic activity, excitatory

## Abstract

The synaptic cleft is the extracellular part of the synapse, bridging the pre- and postsynaptic membranes. The geometry and molecular organization of the cleft is gaining increased attention as an important determinant of synaptic efficacy. The present study by electron microscopy focuses on short-term morphological changes at the synaptic cleft under excitatory conditions. Depolarization of cultured hippocampal neurons with high K^+^ results in an increased frequency of synaptic profiles with clefts widened at the periphery (open clefts), typically exhibiting patches of membranes lined by postsynaptic density, but lacking associated presynaptic membranes (18.0% open clefts in high K^+^ compared to 1.8% in controls). Similarly, higher frequencies of open clefts were observed in adult brain upon a delay of perfusion fixation to promote excitatory/ischemic conditions. Inhibition of basal activity in cultured neurons through the application of TTX results in the disappearance of open clefts whereas application of NMDA increases their frequency (19.0% in NMDA vs. 5.3% in control and 2.6% in APV). Depletion of extracellular Ca^2+^ with EGTA also promotes an increase in the frequency of open clefts (16.6% in EGTA vs. 4.0% in controls), comparable to that by depolarization or NMDA, implicating dissociation of Ca^2+^-dependent trans-synaptic bridges. Dissociation of transsynaptic bridges under excitatory conditions may allow perisynaptic mobile elements, such as AMPA receptors to enter the cleft. In addition, peripheral opening of the cleft would facilitate neurotransmitter clearance and thus may have a homeostatic and/or protective function.

## Introduction

The extracellular space between the pre- and postsynaptic compartments constitutes the synaptic cleft, considered to be the third compartment of the synapse. By electron microscopy (EM), the synaptic cleft is observed to contain electron dense proteinaceous material (Lucic et al., 2005; Zuber et al., 2005; Burette et al., 2012; High et al., 2015; Cole and Reese, 2023), including cell adhesion molecules, extracellular proteins and extracellular moieties of various receptors and ion channels (Missler et al., 2012; Loh et al., 2016; Cijsouw et al., 2018). Trans-synaptic bridges formed through association of cell adhesion molecules from opposite sides of the junction maintain a space of mostly regular width and define the synaptic contact area. Other transsynaptic assemblies aligning synaptic vesicle (SV) release sites to postsynaptic receptors (Martinez-Sanchez et al., 2021) are part of nano columns thought to influence synaptic efficacy (Tang et al., 2016).

Activity induces structural changes at the synapse that may underlie short or long-term modification of synaptic efficacy. Factors controlling synaptic efficacy, the amount of neurotransmitter released from the presynaptic site, and the number and activity of receptors at the postsynaptic site are well established (Thomson, 2000; Nicoll, 2017). An emerging third factor, spatiotemporal availability of neurotransmitter to receptors, brings the synaptic cleft into focus as another crucial site for the control of synaptic efficacy. Concepts such as degree of alignment of SV release sites with receptors regulating micro-domain concentration of neurotransmitter (Raghavachari and Lisman, 2004; Biederer et al., 2017; Chen et al., 2018), geometry of the synaptic cleft regulating neurotransmitter concentration and clearance (Kruk et al., 1997; Savtchenko and Rusakov, 2004, 2007; Burette et al., 2012; Glebov et al., 2016) are gaining interest. While activity-induced ultrastructural changes in the pre- and postsynaptic compartments have been widely documented (Heuser and Reese, 1973, 1981; Dosemeci et al., 2001, 2016; Ostroff et al., 2002; Tao-Cheng, 2006, 2019; Bourne et al., 2013; Harris, 2020; Jung et al., 2021) relatively little is known on activity-induced structural modifications at the synaptic cleft.

Hippocampal neurons in culture offer a suitable, easy to manipulate, experimental tool for the observation of activity-induced structural modifications at the synapse. Our previous EM studies revealed extensive re-organization at the pre- and postsynaptic compartments of cultured hippocampal neurons upon depolarization with high K^+^ or application of NMDA (Dosemeci et al., 2001, 2002, 2013, 2015, 2017; Tao-Cheng, 2006, 2019; Tao-Cheng et al., 2010, 2016; Yang et al., 2011). In the present study, evaluating structural changes at the synaptic cleft, we revisited archived EM data from these previous studies.

## Materials and methods

### Preparation, treatment, fixation and pre-embedding immunogold labeling of rat dissociated hippocampal neuronal cultures

Most images (including depolarization with high K^+^, NMDA, and EGTA experiments) were from previously published reports (Dosemeci et al., 2001; Tao-Cheng, 2006, 2020; Tao-Cheng et al., 2006, 2016) and reexamined here for structural changes of the synaptic cleft. Images of TTX experiments were from archived, unpublished data, and images of recovery after depolarization with high K^+^ experiments were collected from archived grids.

Briefly, cell cultures were prepared from embryonic 20-day-old rat fetuses by papain dissociation, and then plated on glial feeder cultures, and examined at 19–22 days *in vitro* (DIV). Each experiment listed in the present paper was carried out with sister cultures prepared from fetuses of one ‘time-pregnant’ rat, and a typical cell culture preparation would yield eight to twelve 35 mm-dishes of samples. Each culture dish was treated for one experimental condition, resulting in one EM sample which after embedding can be cut up into multiple blocks for sampling.

Culture dishes were placed on a floating platform in a water bath maintained at 37°C. Control incubation medium was HEPES-based Krebs Ringer at pH 7.4. High K^+^ medium was at 90 mM KCl, with osmolarity compensated by reducing the concentration of NaCl. N-methyl-D-aspartic acid (NMDA) medium contained 30–50 μM NMDA in the control medium. APV (an NMDA receptor antagonist) was at 50 μM in control medium. An activity inhibition cocktail was with 100 μM TTX, 50 μM APV and 100 μM CNQX in control medium. EGTA (a calcium chelator) was at 1 mM in Ca^2+^-free medium osmolarity compensated with sucrose. Five sets of experiments were evaluated: cell cultures were washed with control medium and treated for (1) 2 min with control or high K^+^ medium, (2) 2 min with control, NMDA, or APV medium, (3) 1 h in the incubator with control medium or TTX/APV/CNQX medium, (4) 2–5 min with control or EGTA-medium, (5) 2 min with high K^+^ medium, then washed with control medium four times within 2 min and then let to recover in control medium for 30 min. Following incubation, cells were fixed immediately.

For optimal structural preservation, cells were fixed with 4% glutaraldehyde in 0.1 M cacodylate buffer at pH 7.4 for 30 min to 1 h at room temperature and then stored at 4°C. For pre-embedding immunogold labeling, cells were fixed with 4% paraformaldehyde in phosphate buffered saline (PBS) for 30–60 min at room temperature, then washed in PBS and stored at 4°C. Samples for pre-embedding immunogold labeling (Tao-Cheng et al., 2021) were permeabilized/blocked with 0.1% saponin/5% normal goat serum in PBS for 1 h, incubated with primary antibody for 1–2 h, incubated with secondary antibody conjugated to 1.4 nm gold particles (1:250, Nanogold from Nanoprobes, Yaphank, NY) for 1 h, washed in water and silver enhanced (HQ silver enhancement kit, Nanoprobes) to make the small gold particles visible. All steps were carried out at room temperature.

Mouse monoclonal antibody against α-CaMKII (clone 6G9(2), 1:100) (Tao-Cheng, 2020), was from Millipore (Billerica, MA, USA); rabbit polyclonal antibody Shank3 (1:200) (Tao-Cheng et al., 2016) was from Synaptic Systems (Goettingen, Germany, RRID: AB2619862).

### Preparation, treatment and fixation of rat hippocampal organotypic slice cultures

All images were from a previous study (Tao-Cheng et al., 2009) and reexamined here for structural changes at the synaptic cleft. Briefly, the hippocampus was removed from postnatal 6–8 day old rats and cut at 250 μm thickness with a tissue chopper. Slices were placed on a cell culture inserts in six-well culture dishes and used 10–14 days *in vitro* with the dishes on a floating platform in a water bath at 37°C, treated with control medium or 50 μM NMDA for 30 s, 1 and 2 min. Slice cultures within each experiment were prepared from liter mates from one dam. Samples were fixed with 2% glutaraldehyde and 2% paraformaldehyde, or 4% glutaraldehyde in 0.1 N cacodylate buffer at pH 7.4 for 1–3 h at room temperature and then stored at 4°C.

### Perfusion fixation of mouse brains

Images from five perfusion-fixed mouse brains from a previously published report (Tao-Cheng et al., 2007) were reexamined here for structural changes at the synaptic cleft. Briefly, adult male mice, 25–35 g in weight, were deeply anesthetized with isoflurane and perfusion fixed through the heart with 2% glutaraldehyde +2% paraformaldehyde in 0.1 M sodium cacodylate buffer at pH7.4 for two C57 black mice (exp 1), or first perfused with 3.75% acrolein+2% paraformaldehyde in PBS, then followed by 2% paraformaldehyde in PBS for three NIH Swiss mice (exp 2). The time interval starting from the moment the diaphragm was cut to the moment when the outflow from the atrium turned from blood to clear fixative was recorded. Those animals that were successfully perfused within 100 s were classified as “fast” perfusion. For the “delayed” perfusion experiments, calcium- and magnesium-containing PBS was first perfused through the heart for 5 min before the start of the fixative. Neurons were mostly under resting state after fast perfusion, while delayed perfusion fixation promoted an ischemic excitatory state (Tao-Cheng et al., 2007). The perfusion-fixed brains were dissected and vibratomed into 100 μm thick coronal slices and stored in 2% glutaraldehyde in 0.1 M cacodylate buffer at 4°C.

### Electron microscopy

Most samples fixed with glutaraldehyde for structural analysis were post-fixed with 1% osmium tetroxide in 0.1 M cacodylate buffer for 1 h on ice and stained with 1% uranyl acetate in 0.1 N acetate buffer at pH 5.0 overnight at 4°C. Additionally, some samples were post-fixed with “reduced osmium” (1% potassium ferrocyanide +1% osmium tetroxide) in 0.1 M cacodylate buffer for 1 h on ice. Notably, the “reduced osmium” treatment typically does not stain the deeper layer of the PSD as dark as the regular osmium treatment (Tao-Cheng, 2019), and images from these samples were included here for illustration only and not for morphometry.

Samples for immunogold labeling were treated with 0.2% osmium tetroxide in 0.1 M phosphate buffer for 30 min on ice, followed by 0.25% uranyl acetate in acetate buffer at pH 5.0 on ice for 30 min-1 h. Both types of samples (for structural study or for immunogold labeling) were dehydrated in a graded series of ethanol and embedded in epoxy resins. Thin sections were cut at ~70 nm and counterstained with uranyl acetate and lead citrate. Images were photographed on a JEOL 1200 EX transmission electron microscope at 60 KV with a bottom-mounted digital CCD camera (AMT XR-100, Danvers, MA, USA).

### Morphometry

#### Identification of synapses

Identification of synapses was based on criteria described in a classic EM atlas (Peters et al., 1991) and a review (Harris and Weinberg, 2012). The present study focused on glutamatergic excitatory synapses, which are characterized by (1) clusters of SV in presynaptic axonal terminals, (2) the synaptic cleft with a uniform gap of ~20 nm between the pre- and postsynaptic membranes, and (3) the postsynaptic density (PSD) on the dendritic element, facing the active zone of the presynaptic terminal. Sections were screened at magnification of 10,000x for presence of synapses, and then zoomed to magnification of 40,000x for photography.

For perfusion-fixed brains, synapses were sampled from stratum radiatum in CA1 region of the hippocampus within 50 μm of the neuronal soma cluster of the stratum piramidale, and from the outer molecular layer of the cerebellum (Tao-Cheng et al., 2007) with defined types of glutamatergic synapses. Similarly, for organotypic hippocampal slice cultures, glutamatergic synapses were sampled from stratum radiatum in CA1 region proximal to stratum pyramidale (Tao-Cheng, 2019). In contrast, dissociated hippocampal cultures contain a mixture of different types of synapses form all regions of the hippocampus, including CA1, 2, 3 and dentate gyrus. Within randomly chosen grid openings, every excitatory synaptic profile encountered was photographed for sampling until at least five grid openings were examined. Sampling of synapses as well as scoring of synaptic cleft edges were not carried out blind.

#### Scoring of synaptic profiles with ‘open cleft’ at the lateral edges of PSDs

**Figure 1:**
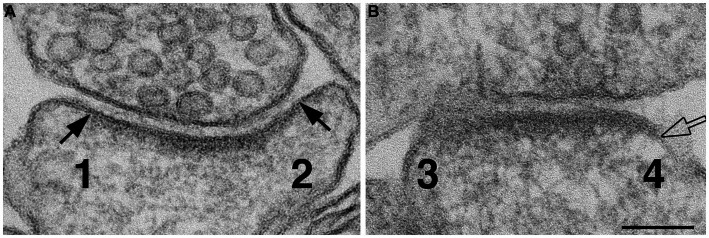
Method for scoring normal vs. ‘open cleft.’ Numbers 1 & 2 in panel **(A)** represent the two edges of a synaptic profile showing cross-sectioned pre- and postsynaptic membranes apposed to each other with a uniform gap throughout the synaptic junctional area. Whereas the synaptic profile in panel **(B)** shows only one cross-sectioned edge (marked by a hollow arrow and labeled as 4). Numbers 1 and 2 are scored as ‘normal,’ number 3 is discarded because it is not cross-sectioned with discernable pre- and postsynaptic membranes, and number 4 is scored as “open” because part of the PSD-lined postsynaptic membrane extends beyond the uniformly spaced region of the cleft. Scale bar = 100 nm.

The lateral edges of cross-sectioned synaptic profiles were scored from sampled images, and there were at least ~40 scored edges in each experimental group for statistical analysis. If the pre- and postsynaptic membranes at the edge of PSD were apposed with the typical uniform gap, this edge was scored as ‘normal’ (marked by solid arrows in Figure 1A). If the distance between the two membranes were wider than the 20 nm gap (hollow arrow in Figure 1B), this edge was scored as ‘open.’ Percentages of ‘open clefts’ were calculated for each sample as the frequency of open clefts.

#### Statistical analysis

Within each experiment, statistical significance on frequency of open clefts was evaluated by Chi-Square test of independence.

## Results

**Figure 2:**
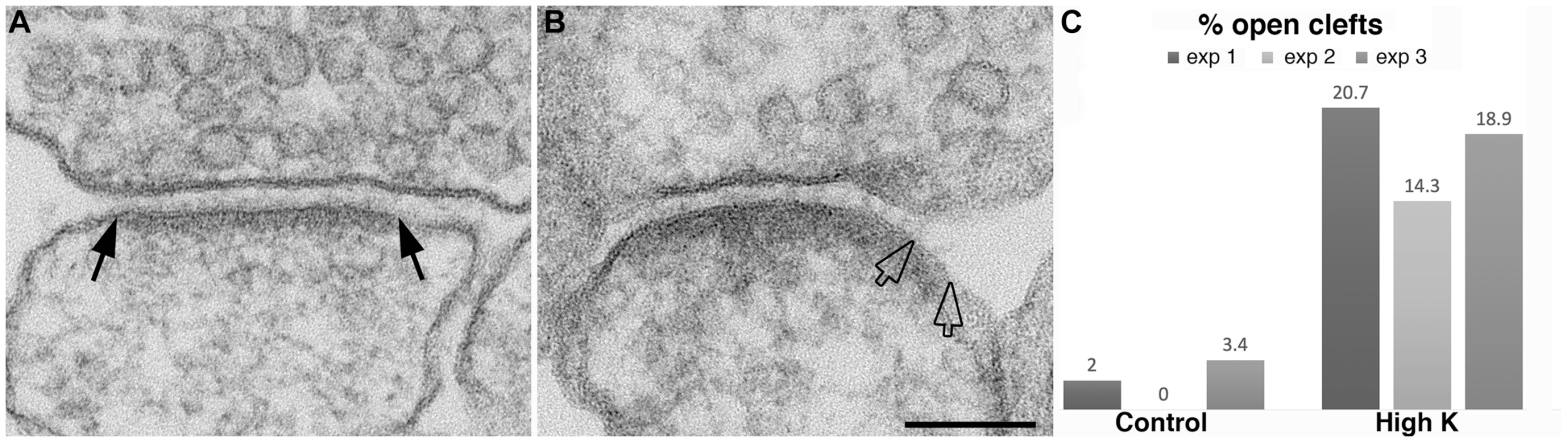
Synaptic profiles from dissociated hippocampal neuronal cultures under control or high K^+^ conditions. **(A)** Under resting conditions, the great majority of synapses contain rigidly apposed pre- and postsynaptic membranes with a uniform cleft width (two solid arrows mark the PSD edges in a synaptic profile with a normal cleft). **(B)** Upon high K^+^ treatment, some PSDs extend beyond the regular cleft area, lining a part of the postsynaptic membrane (marked by hollow arrows) without apposed presynaptic membrane. Scale bar = 100 nm. **(C)** Bar graphs from three experiments.

Hippocampal neurons, 3 weeks in culture, were depolarized for 2 min in medium containing 90 mM K^+^. Electron micrographs of synapses from control and depolarized neurons are shown in Figure 2. Under resting (control) conditions, the presynaptic terminal contains numerous synaptic vesicles (SVs), and the postsynaptic membrane is lined by an electron-dense layer, the postsynaptic density (PSD), typical of glutamatergic excitatory synapses (Figure 2A). Upon depolarization in high K^+^, some depletion of SVs and a marked thickening of the PSD are observed (Figure 2B) as described previously (Dosemeci et al., 2001; Tao-Cheng, 2006).

**Figure 3:**
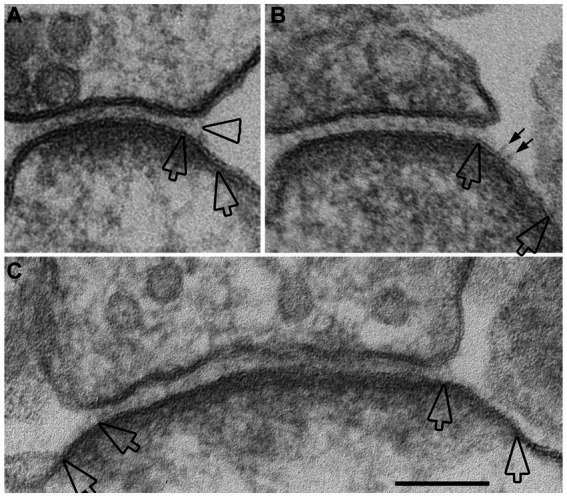
Examples of open clefts in neuronal cultures depolarized with high K^+^. Portions of PSDs that extend beyond the regular cleft area are demarcated by two hollow arrows. **(A)** A delta-shaped opening of pre- and postsynaptic membranes, with synaptic cleft material that appears stretched at the mouth of the cleft (hollow arrowhead). This sample was treated with “reduced osmium.” **(B)** A relatively large stretch of PSD-lined postsynaptic membrane, unattached to a presynaptic counterpart, shows whiskers on the extracellular surface (small arrows) that may represent cleft material. **(C)** A cross-sectioned synaptic profile exhibiting open clefts at both edges. Scale bar = 100 nm.

A closer examination of the cleft regions from control and depolarized samples revealed a hitherto unnoticed phenomenon. While in the majority of synapses the cleft presents a fairly uniform width throughout the contact area (Figure 2A), in a number of synapses, the cleft appears to widen or open up at the periphery. In these ‘open clefts,’ the PSD-lined membrane (area between two hollow arrows in Figures 2B, 3) is no longer apposed by the presynaptic membrane. This type of open cleft is encountered more readily in depolarized samples. A quantitative evaluation of the frequency of open clefts in synapses in control and high K^+^ media reveals a significant increase upon depolarization (Figure 2C; Table 1). The degree of opening at the periphery of synaptic cleft varies, from a delta-shaped opening in some (Figure 3A), to an extensive area of PSD-lined postsynaptic membrane unopposed by presynaptic membranes in others (Figures 3B,C). When depolarized cultures were returned to control Ringer medium for 30 min, the frequency of open clefts decreased to near control levels, indicating that peripheral widening of the synaptic cleft during activity is a transient phenomenon (Table 2).

**Table 1:** Frequency (%) of open clefts in synaptic profiles of dissociated cultures under different conditions.

	Control	High K^+^	NMDA	APV	TTX/APV/CNQX	EGTA
Exp 1	2% (50)	20.7% (58) *p* < 0.005				
Exp 2	0% (42)	14.3% (70) *p* < 0.05				
Exp 3	3.4% (87)	18.9% (106) *p* < 0.005				
**Mean ± SEM**	**1.8 ± 1.0**	**18.0 ± 1.9**				
Exp 1	5.2% (155)		12.4% (89) *p* < 0.05			
Exp 2	5.3% (57)		19.5% (41) *p* < 0.05 vs. control	3.1% (64) *p* < 0.01 vs. NMDA		
Exp 3			25% (44)	2.1% (48) *p* < 0.005		
**Mean ± SEM**	**5.3 ± 0.1**		**19.0 ± 3.6**	**2.6 ± 0.5**		
Exp 1	3.6% (55)				0 (44) *p* = 0.2	
Exp 2	4.4% (45)				0 (40) *p* = 0.18	
Exp 3	3.8% (78)				0 (74) *p* = 0.09	
**Mean ± SEM**	**3.9 ± 0.2**				**0**	
Exp 1	3.8% (131)					16.0% (169) *p* < 0.001
Exp 2	2.3% (44)					17.3% (52) P < 0.05
Exp 3	5.5% (55)	20% (40) *p* < 0.05 vs. control				16.4% (67) *p* = 0.06 vs. control
**Mean ± SEM**	**4.0 ± 1.1**					**16.6 ± 0.4**

*P* values of Chi-Square test of independence within each experiment are listed in each row.

(n) = number of clef edges scored.

**Table 2:** Frequency (%) of open clefts from sister cultures under control, high K^+^ and recovery conditions.

	Control	High K^+^	2′ K^+^ + 30′ recovery
Exp 1	1.8% (167)	26.0% (77) *p* < 0.0001 vs. control	6.1% (114) *p* < 0.005 vs. high K^+^
Exp 2	4.4% (136)	17.1% (105) *p* < 0.005 vs. control	1.7% (58) *p* < 0.005 vs. high K^+^

Statistical significance within each experiment evaluated by Chi-Square test of independence.

(n) = number of cleft edges scored.

**Figure 4:**
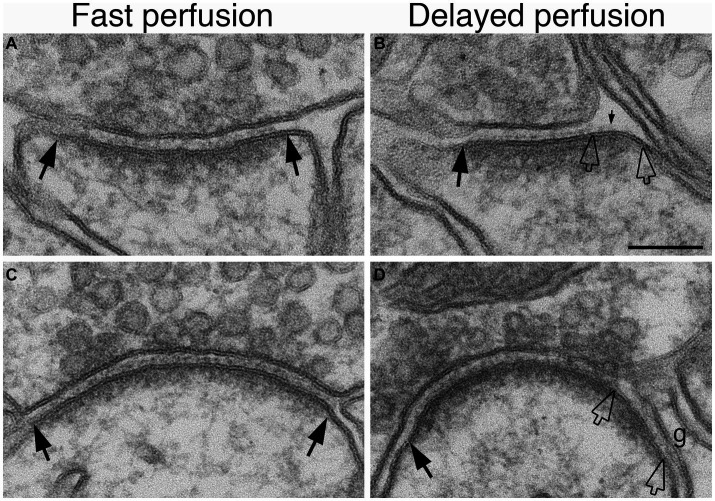
Examples of normal and open clefts from perfusion-fixed brains. Normal clefts are marked by solid arrows. In open clefts, PSD areas lacking apposed presynaptic membrane are demarcated by two hollow arrows. More open clefts are observed in delayed perfusion-fixed mouse brains **(B,D)** than in fast perfusion-fixed brains **(A,C)**. Top row is sampled from the stratum radiatum of the CA1 region of the hippocampus, and the bottom row is from molecular layer of the cerebellum. Notice that in panel **(D)** a glia process (g) is covering this open cleft. Scale bar = 100 nm.

We had previously observed that delayed perfusion fixation promotes excitatory conditions in brain that induces thickening of the PSD (Tao-Cheng et al., 2007). Thus, we re-examined specimens from fast and delayed perfusion fixation protocols to find out whether excitatory conditions also induce cleft opening in the adult brain. Figure 4 illustrates synapses from two regions of mouse brain, hippocampus (upper panels) and cerebellum (lower panels), upon fast (Figures 4A,C) vs. delayed (Figures 4B,D) perfusion fixation. In both regions, there were consistently more open clefts in delayed than in fast perfusion-fixed brains (Table 3). Notably, glial processes occasionally were seen covering the PSD-lined membranes in these open clefts (Figure 4D).

**Table 3:** Frequency (%) of open clefts from two different regions of mouse brain under fast or delayed perfusion fixation conditions.

	Fast perfusion fixation	Delayed perfusion fixation
Hippocampus CA1, stratum radiatum
Exp 1	1.2% (81)	21.4% (140) *p* < 0.0001
Exp 2	20% (65)	30.4% (46) (not significant)
Cerebellum molecular layer
Exp 1	5.6% (36)	27.8% (79) *p* < 0.01
Exp 2	20.6% (102)	37.3% (48) *p* < 0.05

Statistical significance within each experiment analyzed by Chi-Square test of independence, and listed in each row.

(n) = number of cleft edges scored.

**Figure 5:**
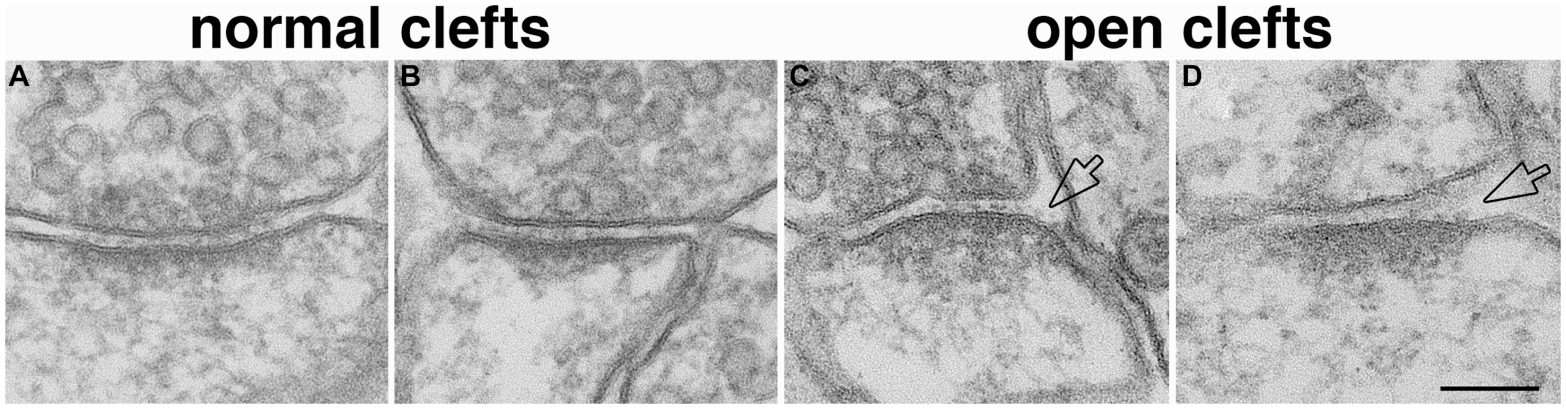
Open clefts are often associated with PSDs of increased thickness and curvature. **(A,B)** Normal clefts sampled from fast perfusion-fixed hippocampus, where PSDs are often with a concave **(A)** or flat **(B)** curvature. **(C,D)** Open clefts (hollow arrows) sampled from delayed perfusion-fixed hippocampus, where PSDs are thicker and often with a convex **(C)** curvature. Scale bar = 100 nm.

Perfusion-fixed brain, with well-defined glutamatergic synapses in specific regions, offers a suitable system to assess whether open clefts are associated with special feature of the PSD. It has been shown that delayed perfusion fixation did not change the length of PSD in these brain regions, but significantly increased the thickness and curvature of the PSD (Tao-Cheng et al., 2007). In two delayed perfusion-fixed hippocampi, ~ 90% (40 out of 44) of open clefts were associated with conspicuously thickened PSDs (Figures 5C,D). Of these open clefts, 84% were associated with PSDs with a convex curvature, where PSDs appeared bending toward the center of the spine (Figure 5C), 17% with flat PSDs (Figure 5D), and virtually no open clefts were seen associated with PSDs with a concave curvature. In contrast, in two fast perfusion-fixed hippocampi, the great majority (85%, 132 out of 156) of clefts was scored as normal (Figures 5A,B). Within these normal clefts, the relative frequencies of PSD curvature types were: 42% concave (Figure 5A), 46% flat (Figure 5B), and 12% convex. It should be noted that within our restricted sampling region in the hippocampus, the great majority (>90%) of PSDs were macula (Nicholson et al., 2006) which are smaller in size (~190 nm in average PSD length from single sections, Tao-Cheng et al., 2007) than perforated PSDs (Harris and Weinberg, 2012). Although open clefts were observed in both types of PSDs, due to the low number of perforated PSDs in our sampling, a quantitative evaluation of possible differences between the two types of PSDs could not be carried out.

As noted above (Table 2), when depolarized cultures are returned to normal medium, the frequency of open clefts decreases to basal levels, indicating that the observed change is transient. Yet, the frequency of synapses with open clefts in control samples from cultures, and in rapidly fixed brains is not zero, and, in the case of fast perfusion-fixed brains, highly variable. A pertinent question is whether those open clefts observed under ‘basal’ conditions represent a different ‘sustained’ category. Alternatively, it is possible that open clefts under basal conditions are due to variable levels of excitatory neurotransmitter release, caused by spontaneous activity in the cultures, and ischemia upon cessation of blood flow during perfusion fixations.

**Figure 6:**
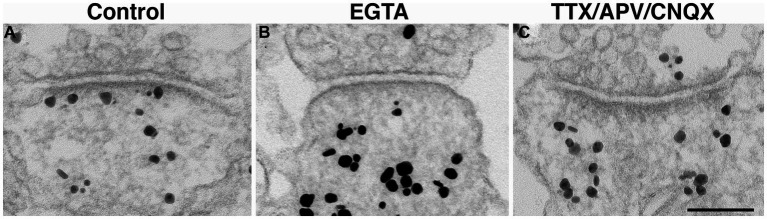
Synaptic profiles of hippocampal neuronal cultures labeled with CaMKII. CaMKII distribution is a reliable indicator of the calcium levels within the spine, and thus, an indicator of the activity state of the synapses (Tao-Cheng, 2020). Under control conditions, label for CaMKII is typically dispersed in the cytoplasm with some associated with the PSD **(A)**. In contrast, under calcium-free conditions (EGTA medium for 5 min), many PSDs lack CaMKII labeling **(B)**. Upon 1 h incubation with TTX/APV/CNQX, many PSD are also devoid of CaMKII labeling **(C)**, indicating low levels of postsynaptic calcium. Scale bar = 100 nm.

Because activity levels cannot be easily controlled during perfusion fixation (Tao-Cheng et al., 2007), we chose to use neuronal cultures to further test the involvement of activity in the occurrence of open clefts under basal conditions. Neuronal cultures were treated with an inhibitory cocktail containing TTX (sodium channel blocker), APV (NMDA receptor antagonist) and CNQX (AMPA receptor antagonist) for 1 h to block spontaneous activity. Although the Chi-Square test of independence within individual experiments did not reach statistical significance, data from three experiments showed no open clefts in TTX/APV/CNQX-treated samples compared to 3.6, 4.4 and 3.8% of open clefts in matching controls (Table 1). Additional evidence from cultures labeled with CaMKII, whose distribution at the postsynaptic compartment is a reliable indicator of the activity state of glutamatergic synapses (Tao-Cheng, 2020), further confirmed that spontaneous activity was indeed blocked by the cocktail (Figure 6), and this lack of activity may be linked to the absence of open clefts.

**Figure 7:**
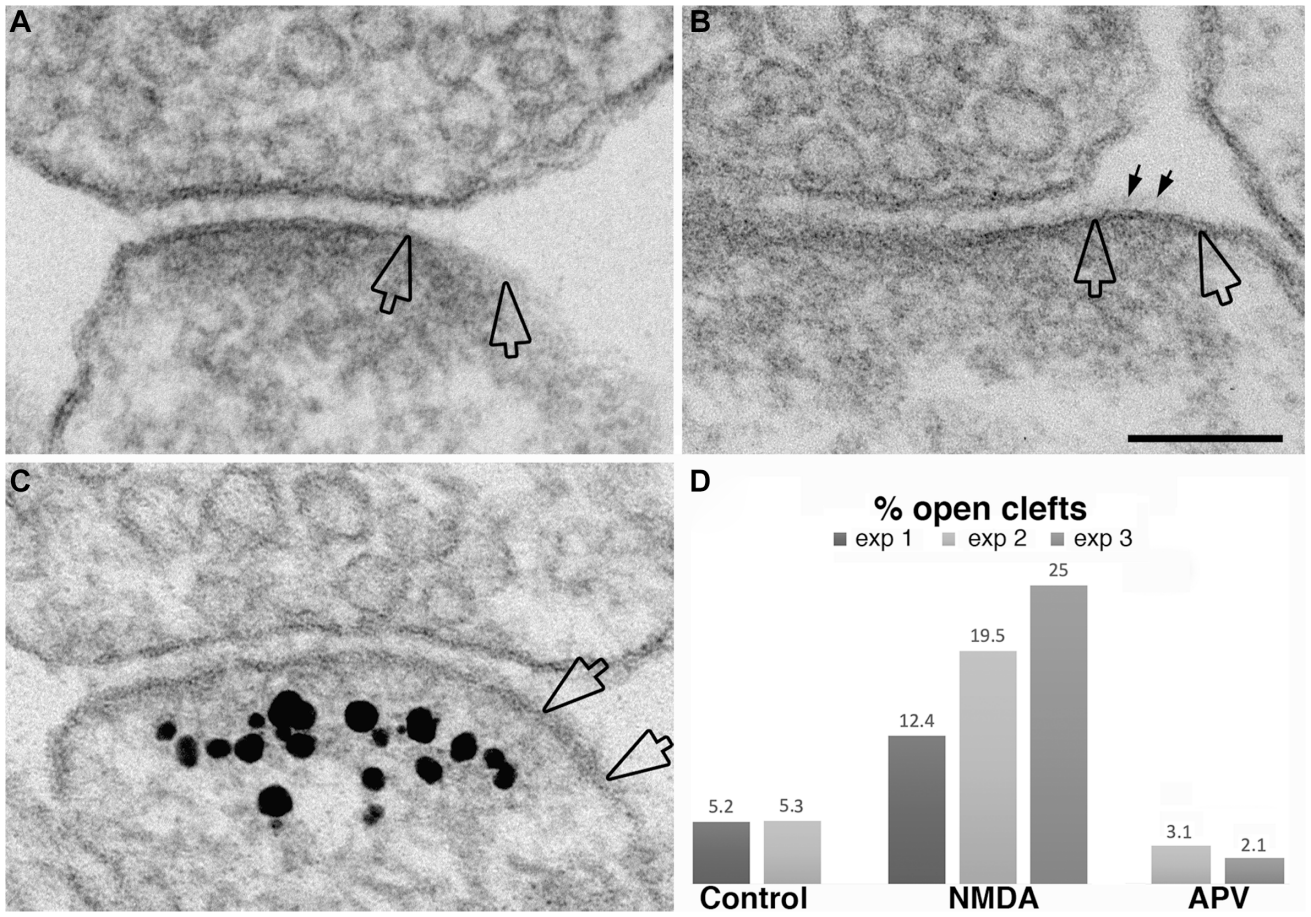
Examples of open clefts in NMDA-treated neuronal cultures. PSD areas lacking apposed presynaptic membrane are marked between two hollow arrows in panel **(A–C)**. Some cleft material (small arrows in **B**) is visible on the exposed PSD-lined postsynaptic membrane. **(C)** Was labeled for Shank3, a PSD scaffold protein (Tao-Cheng et al., 2016). Scale bar = 100 nm. **(D)** Bar graphs from three experiments.

While the data presented above established that synaptic activity causes the appearance of open clefts, the underlying mechanism of this phenomenon remained to be clarified. Indeed, changes at the cleft could be initiated through either pre- or postsynaptic signals. One strategy to dissect postsynaptic receptor activation from presynaptic neurotransmitter release is direct application of neurotransmitter agonists. We investigated whether activation of NMDA receptors could mimic the effects of depolarization in promoting the appearance open clefts. Figure 7 illustrates synaptic profiles from NMDA-treated samples, showing clefts opened up in varying degrees, as judged by the length of the PSD-lined postsynaptic membrane, without apposed presynaptic membrane. Figure 7C shows one example of PSD immunogold labeled for Shank3, a PSD scaffold protein. Quantitative assessment indicates that application of 30–50 μM NMDA for 2 min results in an increase in the frequency of open clefts (Figure 7D; Table 1).

Organotypic hippocampal cultures offer yet another experimental system, one that retains the original anatomical organization of hippocampal neurons, to study structural changes at the synaptic cleft under pharmacological manipulations. Sampling from stratum radiatum of the CA1 region from organotypic cultures, we tested whether the frequency of open clefts increases with increasing exposure time to NMDA. Data from specimens corresponding to 0.5, 1, and 2 min NMDA exposure intervals indeed show a gradual increase in the frequency of open clefts with exposure time and indicates that activation of NMDA receptors sustained for a minute or more is required for the appearance of significant numbers of open clefts (Table 4).

**Table 4:** Frequency (%) of open clefts in organotypic hippocampal slice cultures treated with NMDA (50 μM) at different time points.

	Control	30 s NMDA	1 min NMDA	2 min NMDA
Exp 1	5.1% (99)	8.1% (86)	15.7% (102) *p* < 0.05 vs. control	32.9% (79) *p* < 0.0001 vs. control, *p* < 0.0001 vs. 30 s NMDA, *p* < 0.01 vs. 1 min NMDA
Exp 2	5.8% (103)	6.9% (72)	13% (100) *p* < 0.05 vs. control	25.8% (89) *p* < 0.0001 vs. control, *p* < 0.001 vs. 30 s NMDA, *p* < 0.05 vs. 1 min NMDA

Statistical significance within each experiment evaluated by Chi-Square test of independence.

(n) = number of cleft edges scored.

**Figure 8:**
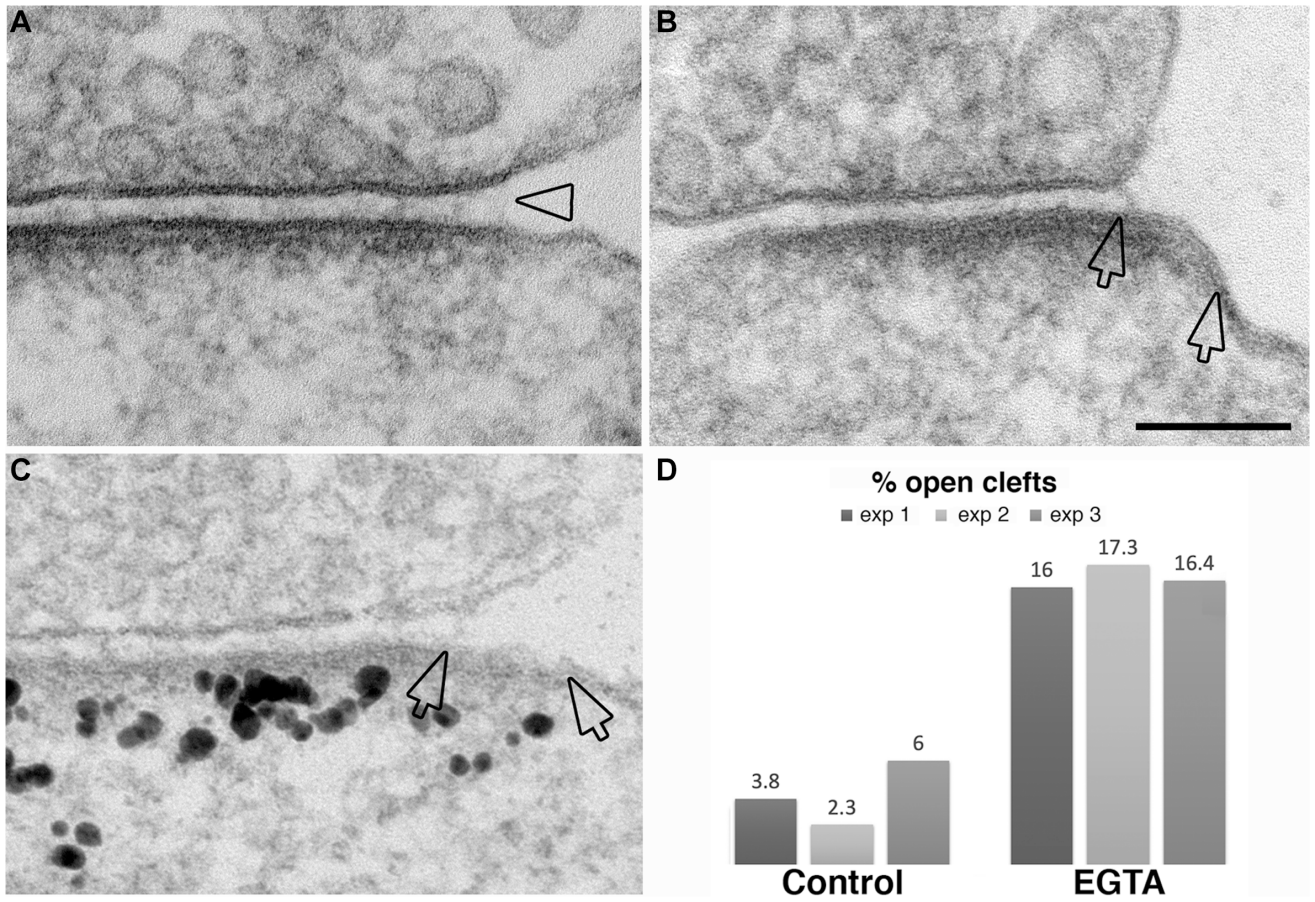
Examples of open clefts in EGTA-treated neuronal cultures. **(A)** Shows a subtle separation of the pre- and postsynaptic membranes with cleft material appearing stretched at the mouth (hollow arrowhead). **(B)** Shows an area (between two hollow arrows) of exposed PSD-lined membrane. **(C)** Was labeled for Shank3, a PSD scaffold protein. Two hollow arrows mark the PSD area, labeled with Shank3, lacking apposed presynaptic membrane. Scale bar = 100 nm. **(D)** Bar graphs from three experiments.

The observation that NMDA application induces the appearance of open clefts, indicates that this process is initiated through a postsynaptic stimulus. The next question was whether the observed phenomenon involves dissociation of trans-synaptic bonds between corresponding pre- and postsynaptic partners. It has been reported that interactions between certain synaptic adhesion molecules, including N-cadherin to N-cadherin and neuroligin to neurexin are partially calcium dependent (Missler et al., 2012). We reasoned that removal of calcium from the extracellular medium could specifically target these transsynaptic associations. Neuronal cultures incubated in calcium-free medium containing 1 mM EGTA for 2–5 min indeed exhibited open clefts (Figures 8A–C) with frequencies consistently higher than those in control cultures in three experiments (Figure 8D; Table 1). Notably, PSDs in these EGTA-treated samples were not thickened like those treated with high K^+^ (Figures 2, 3) or NMDA (Figure 7), suggesting a different mechanism.

## Discussion

Excitatory conditions, including depolarization with high K^+^ and application of NMDA in neuronal cultures, and delayed perfusion fixation in adult brain, promote the appearance of open clefts, a widening at the periphery of the synaptic cleft. Some synaptic profiles show delta shaped openings, while, in others, long stretches of PSD-lined membranes are visible at the postsynaptic side, lacking an apposed presynaptic membrane. Occasionally elongated transsynaptic bridges can be seen at the mouth of the widening, suggesting conformational changes of adhesion molecules (Zhou, 2023). Occasional whisker-like material on the extracellular side of PSD-lined membranes could be dissociated adhesion molecules or other cleft components such as receptors.

Because the PSD typically demarcates the synaptic contact area at the postsynaptic side, the appearance of PSD lined membranes without apposed presynaptic membranes at the peripheries of the cleft implies dissociation of transsynaptic bridges at these regions. An alternative interpretation, activity-induced rapid lateral expansion of the PSD through addition of new materiel to the periphery, does not seem likely. Although lateral expansion of the PSD has been described upon LTP-inducing stimuli, this phenomenon is latent, appearing about 2 h after LTP induction (Ostroff et al., 2002; Chirillo et al., 2019). Thus, for the rest of the discussion we shall define the observed modification as dissociation of peripheral transsynaptic bridges.

Under any of the conditions tested, only a fraction, up to 20%, of all synaptic profiles show a peripheral dissociation at the cleft, implying a selective phenomenon. It is possible that these profiles belong to a specific morphological type of synapses. Alternatively, in any one synapse, only certain areas of the cleft periphery may be opening up, so that the cross-section profiles do not always catch the affected region. Further studies by serial section EM should clarify the relative contributions of these two scenarios. A previous study by EM serial section and tomography (Bell et al., 2014) describes “nascent zones” at the periphery of the synapse that share a PSD with the active zone but lack synaptic vesicles. Whether the peripheral widened cleft parts observed in the present study correspond to these nascent zones remains an open question also to be resolved by detailed three dimensional analyses.

Selective dissociation of the cleft at the peripheries under excitatory conditions implies localization of susceptible transsynaptic bridges in those specific regions. The observation that depletion of Ca^2+^ by the application of EGTA also induces similar frequencies of open clefts suggests that these susceptible transsynaptic bridges may be those inter-molecular associations that are Ca^2+^ dependent. However, as the reduction of extracellular Ca^2+^ has been described to increase neuronal excitability (Lu et al., 2010), the contribution of an indirect mechanism, through EGTA-induced excitation, cannot be excluded for the observed changes in cleft morphology.

When considering the functional implications of peripheral dissociation of the cleft under excitatory conditions, an initial question is whether it is a temporary or permanent event. Data presented in Table 2 establishes that upon cessation of excitatory conditions, the frequency of synaptic profiles with open clefts returns to near control levels. Moreover, suppression of basal activity with TTX and glutamate receptor antagonists causes total disappearance of open clefts. These data indicate that cleft opening is not maintained in the absence of activity.

What would be the functional consequences of a temporary disruption of transsynaptic bridges under excitation? Theoretical simulations predict that the geometry of the synaptic cleft would influence synaptic efficacy by controlling the concentration and clearance of neurotransmitter (Kruk et al., 1997; Savtchenko and Rusakov, 2004, 2007). A previous study (Glebov et al., 2016) suggested an increase in synaptic efficacy through a 1.1 nm uniform narrowing of the cleft upon long-term application of TTX and, another EM study (Burette et al., 2012), described constrictions at the edges of the cleft that would inhibit neurotransmitter clearance. The present observation of peripheral opening of the cleft during activity may allow rapid clearance of neurotransmitter out of the cleft and thus may have a homeostatic (Turrigiano, 2012) or protective effect by preventing neurotransmitter buildup. Rebuilding of transsynaptic bridges upon cessation of activity would restore basal synaptic efficacy. In addition to possible homeostatic functions, peripheral disruption of transsynaptic bridges may also offer a window of plasticity for the re-organization of cleft elements at these locations. Of particular interest for synaptic plasticity is the lateral diffusion of AMPA receptors in the plasma membrane to and from postsynaptic sites (Borgdorff and Choquet, 2002). Temporal disruption of transsynaptic bridges in those peripheral regions would facilitate exchange of receptors between the peri-synaptic and PSD-associated pools.

## Data availability statement

The original contributions presented in the study are included in the article/supplementary material, further inquiries can be directed to the corresponding authors.

## Ethics statement

The animal study was approved by National Institute of Neurological Disorders and Stroke/National Institute of Deafness and Communication Disorders/National Center for Complementary and Integrative Health Animal Use and Care Committee. The study was conducted in accordance with the local legislation and institutional requirements.

## Author contributions

J-HT-C, SM, CW, and AD were involved in the performance of experiments. J-HT-C collected EM data. J-HT-C and AD designed the experiments, evaluated the data, and wrote the manuscript. All authors were involved in the interpretation of results and discussions of the manuscript.

## Funding

This study was supported by NINDS intramural funds.

## Conflict of interest

The authors declare that the research was conducted in the absence of any commercial or financial relationships that could be construed as a potential conflict of interest.

## Publisher’s note

All claims expressed in this article are solely those of the authors and do not necessarily represent those of their affiliated organizations, or those of the publisher, the editors and the reviewers. Any product that may be evaluated in this article, or claim that may be made by its manufacturer, is not guaranteed or endorsed by the publisher.
